# Control of Microalgae Growth in Artificially Lighted Photobioreactors Using Metaheuristic-Based Predictions

**DOI:** 10.3390/s21238065

**Published:** 2021-12-02

**Authors:** Viorel Minzu, George Ifrim, Iulian Arama

**Affiliations:** 1 Control and Electrical Engineering Department, “Dunarea de Jos” University, 800008 Galati, Romania; george.ifrim@ugal.ro; 2 Informatics Department, “Danubius” University, 800654 Galati, Romania; iulian.arama@univ-danubius.ro

**Keywords:** optimal control problem, closed-loop control structure, optimal predictions, microalgae growth model, soft sensors, adaptive particle swarm optimization

## Abstract

A metaheuristic algorithm can be a realistic solution when optimal control problems require a significant computational effort. The problem stated in this work concerns the optimal control of microalgae growth in an artificially lighted photobioreactor working in batch mode. The process and the dynamic model are very well known and have been validated in previous papers. The control solution is a closed-loop structure whose controller generates predicted control sequences. An efficient way to make optimal predictions is to use a metaheuristic algorithm, the particle swarm optimization algorithm. Even if this metaheuristic is efficient in treating predictions with a very large prediction horizon, the main objective of this paper is to find a tool to reduce the controller’s computational complexity. We propose a soft sensor that gives information used to reduce the interval where the control input’s values are placed in each sampling period. The sensor is based on measurement of the biomass concentration and numerical integration of the process model. The returned information concerns the specific growth rate of microalgae and the biomass yield on light energy. Algorithms, which can be used in real-time implementation, are proposed for all modules involved in the simulation series. Details concerning the implementation of the closed loop, controller, and soft sensor are presented. The simulation results prove that the soft sensor leads to a significant decrease in computational complexity.

## 1. Introduction

Microalgae biotechnology has attracted increased interest in recent years due to its potential to be used in a wide range of applications. Microalgae can be used in human and animal nutrition and also in the production of a wide range of added-value compounds [1,2]. They can also be involved in environmental applications such as the treatment of wastewater or the production of biofuels. In addition, their capacity to biomitigate carbon dioxide makes microalgae cultivation one of the most eco-friendly biotechnologies.

Light energy is required to perform water photolysis, one of the most energy-demanding reactions in nature. Photosynthetic growth is very attractive due to its simple and cheap requirements. Microalgae may be cultivated in photobioreactors that have at least one transparent side through which the light radiates the culture [3].

This work is focused on controlling microalgae growth in a specific artificially lighted photobioreactor (PBR). Therefore, dynamic models for photobioreactors and control approaches have been of interest to us. Many references concerning microalgae cultures present reliable mathematical models validated with experimental data and propose control strategies [4,5,6].

Light is the main factor that restricts the photosynthetic growth process. It creates a heterogenous light field inside the photobioreactor. The microalgae growth rate decreases according to the depth of the culture [7,8,9]. Thus, these light-driven processes must couple the radiative model with the growth kinetics, resulting in a class of models able to express the specific growth rate as a function of the available light inside the culture (i.e., the irradiance) [4,7].

Discontinuous photobioreactors are artificially lighted for increased productivity and high repeatability of results. The light sources have become more efficient (e.g., LED grow light panels) and easier to control, which has opened up a new class of controllers named lumostats [10,11]. These controllers modify the incident light intensity to control variables such as light-to-microalgae ratio, biomass yield on light energy, etc. General aspects concerning the optimization of microalgae cultures are presented in [6,12,13].

This work has great practical relevance because it is firstly addressed to the practicing professional engineer who wants to implement an optimal control structure for a PBR similar to that described in this paper. Many algorithms and their implementation could be used in a future real-time application.

Nevertheless, the main contribution of this paper is a soft sensor, which is integrated into the control structure and could have theoretical repercussions. The sensor aims to reduce the admissibility domain of the process’s control input according to its current state [14,15]. The admissibility domain contains all the control input values that lead to a favorable process evolution (e.g., meeting certain dynamic constraints, such as the positive growth rate of microalgae). A smaller admissibility interval involves a certain reduction in computational complexity. For the microalgae growth process, the soft sensor proved its utility. The general theoretical idea is that the sensor could be involved in controlling other kinds of processes.

More precisely, several requests and preliminaries define our work:

- A specific artificially lighted PBR for microalgae growth has to be controlled over a given control horizon. This batch PBR is described in [10,11], and sufficient details are given in Section 2 and Appendix A.
- The dynamic process model (PM) concerning the microalgae growth is presented and validated in [10,11]. Section 2 recalls the basic elements that led to this PM used in our work.
- A bi-criteria optimal control problem concerning the PBR at hand is stated in Section 3.1. The optimum criterion requires minimizing the amount of light (the energy) while a specified quantity of biomass is produced.
- The optimal control problem must be solved through a closed-loop control structure. Only in this way will the controller’s output depend on the real state of the PBR (e.g., the biomass concentration) in each sampling period.

The closed-loop control structure proposed in this work is the Receding Horizon Control (RHC) structure [16,17], a general and efficient control structure involving a prediction technique. A module of the Controller, called the *Predictor*, generates sequences of control output values, called predicted control sequences (*pcs*), that will determine the process’s optimal trajectories. This solution entails two other options:

- Generating an optimal *pcs* requires a significant computational effort, as in our control problem. For this reason, the *Predictor* will resort to a metaheuristic algorithm [18,19] that can cope with high computational complexity. An adaptive version of the hybrid topology particle swarm optimization [20,21] algorithm will be integrated into the *Predictor*.
- Despite the ability of the particle swarm optimization algorithm to efficiently generate optimal predictions, we propose a soft sensor to further decrease the computational complexity, especially for very large prediction horizons.

Section 3 states the optimal control problem with all its defining elements and describes the RHC structure and the prediction sequences in the optimality context. Section 3.4 is devoted to the proposed soft sensor and presents its necessity, structure, functioning, and implementation.

To keep the presentation self-contained, Section 4 briefly describes PSO and its advances, namely, hybrid topology and adaptation of particle speed. To conduct the simulation study presented in Section 5, we implemented the proposed algorithms using the MATLAB language and system. Many algorithms were implemented realistically, like in a real-time application. Section 6 presents and discusses the simulation results. These results prove that the control solutions and algorithms work well and that the soft sensor makes a real contribution.

## 2. Photosynthetic Growth of Microalgae: The Process Dynamic Model

The objective of this section is to specify the PBR’s dynamic model, which will be used in the following sections to state and solve a specific optimal control problem. The basic aspects of the considered process model and the PBR’s parameters have already been presented and validated (see [10,11] and Appendix A).

The microalgae are cultivated in photobioreactors of various geometries with transparent regions allowing light to penetrate the culture. The incident light intensity $q_{0}$ is measured at the point where the light touches the microalgae culture. It is attenuated inside the PBR, with the available light for any depth, z, of the culture being named irradiance G(z). Models that describe the attenuation of light inside microalgal cultures are named radiative models.

**Figure 1:**
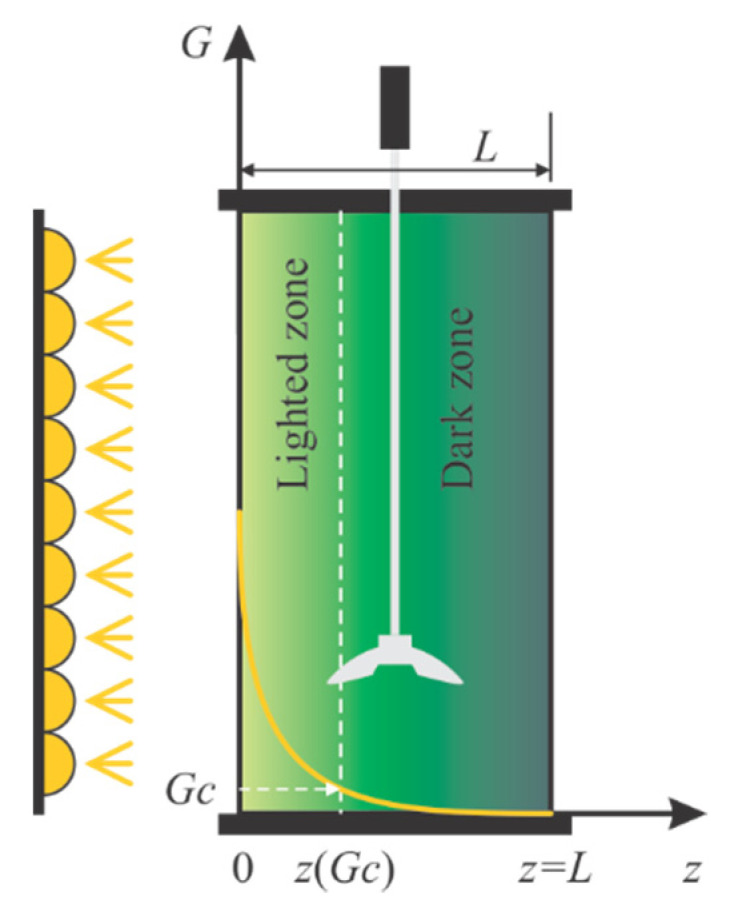
Schematic representation of light attenuation inside the photobioreactor.

This work considers a continuously stirred flat-plate PBR lighted on one side, already presented in [10,11]. The radiative model considered here was presented in [7]. The irradiance is related to the biomass concentration (denoted as *X* for the moment), the depth of the culture (*z*, see Figure 1), and few coefficients: the mass absorption ($E_{a}$), the mass scattering ($E_{S}$), and the backward scattering fraction (*b*).

(1) $$G\left(z\right)=q_{0}\times e^{-\frac{1+\alpha }{2\alpha }E_{a}\times X\times z};\;\alpha =\sqrt{E_{a}/\left(E_{a}+2bE_{s}\right)},$$

where α is the linear scattering modulus.

The attenuation of light creates a heterogenous light field; the growth decreases along with the increase in the culture’s depth. Coupling between radiative models and growth kinetics models is present in many papers and has been investigated by numerous authors. It has been agreed that light is the most important factor that governs growth and must be considered a substrate. Thus, the specific growth rate of microalgae, μ, is a function of the light available inside the culture: μ(G(z)). Because there is a different μ for any z, the coupling between the radiative and kinetic models can be done in two ways:

- An average irradiance, $G_{avg}$, can be computed and used to calculate the specific growth rate, $\mu \left(G_{avg}\right)$;
- Various local photosynthetic responses can be calculated μ(G(z)) for various depths z and used to obtain the average photosynthetic response $\bar{\mu }$.

In this paper, the second approach is considered, even though both approaches give similar results. An inhibition model gives the average specific growth rate:

(2) $$\bar{\mu }=\mu_{max}\frac{1}{L}\overset{L}{\underset{0}{\int }}\frac{G\left(z\right)}{K_{S}+G\left(z\right)+G{\left(z\right)}^{2}/K_{I}}dz\;,$$

where $\mu_{max}$ is the maximum specific growth rate, $K_{S}$ is the saturation constant, and $K_{I}$ is the inhibition constant. L is the depth of the PBR (z∈[0 L]).

The specific growth rate represents the ratio between the newly formed biomass and the existing one, thus being given in h^−1^. To express the biomass concentration, we considered the volumetric growth rate given below:

(3) $$r_{x}=r_{xp}-r_{xd}=\bar{\mu }\times X-\mu_{d}\times X,$$

$r_{x}$ is the volumetric growth rate that balances growth (i.e., $r_{xp}$ = the photosynthetic growth rate) and decay (i.e., $r_{xd}$ = the volumetric decay rate). $\mu_{d}$ is the specific decay rate, which describes processes such as cell death or respiration (opposite to photosynthesis). $\mu_{d}$ is considered constant here.

Batches are done regularly at a constant incident light intensity. Still, many experimental setups allow light variation over a wide range of intensities. In these conditions, by expressing the specific growth rate as a function of light, the biomass is an explicit function of the input, $q_{0}$.

In this paper, we have adopted the hypothesis that the incident light intensity is not constant over the entire control horizon and consequently is denoted by q(t). We emphasize that q(t), which will replace $q_{0}$, is the control input for the BPR considered in the closed-loop control structure proposed in this paper.

The amount of light consumed in the process is calculated by simply integrating the incident light intensity over the interval $\left[t_{0},t\right]$:

$$\mathrm{amount}\;\mathrm{of}\;\mathrm{light}=A\times \int_{t_{0}}^{t}q(\tau )d\tau ,$$

where A is the lighted surface of the reactor.

In this work, we have considered the PBR dynamic model to be the following two ordinary differential equations:

$${\dot{x}}_{1}(t)=r_{x}$$

$${\dot{x}}_{2}(t)=A\times q(t),$$

where $x_{1}(t)$ is the biomass concentration (in g/L^−^) and $x_{2}(t)$ the amount of light consumed up to moment *t* (in µmol/m^2^/s). The nonlinear character of the first differential equation will be clearly expressed in Section 3.1.

The efficiency of a batch can be evaluated by the variable “biomass yield on light energy”, denoted by Y. It is defined by the ratio between the newly produced biomass and the amount of light used in the considered interval:

(4) $$Y(t)=\frac{V\times \left[x_{1}(t)-x_{1}(t_{0})\right]}{x_{2}(t)},$$

where $x_{1}(t_{0})$ is the biomass concentration at the beginning of the batch and V is the working volume of the PBR. Other PBR parameters, such as the specific growth rate and existing biomass, will complete the dynamic model.

## 3. Closed-Loop Control Problem

### 3.1. Optimal Control Problem

The elements defining an optimal control problem (OCP) and their integration into real applications are presented in [22,23]. In the following, we describe the OCP—called the photobioreactor optimal control (PBROC)—that was solved in this work. The three parts that define the PBROC problem are given hereafter.

(1) The dynamic model of the optimized process

As stated in Section 2, the PBR depth is divided into equal $k_{L}$ segments (in our implementation, $k_{L}$ = 100 for *L* = 4 cm). The corresponding depth values are $z_{i},\;i=1,\ldots ,k_{L}$. This discretization involves the calculation of the following constants (see Equation (1)) used for the calculation of the state variables:

(5) $$k_{i}=e^{-\frac{1+\alpha }{2\alpha }\times E_{a}\times z_{i}};\;i=1,\ldots ,k_{L}$$

The PBR’s dynamic model consists of two state equations as follows:

(6) $${\dot{x}}_{1}(t)=r_{x}$$

(7) $${\dot{x}}_{2}(t)=A\times C\times q(t)$$

where

(8) $$\begin{array}{l} r_{x}=(\bar{\mu }-\mu_{d})\times x_{1}(t)\\ G_{i}(t)=q(t)\times k_{i}{}^{x_{1}(t)},\;i=1,\ldots ,k_{L}\\ \bar{\mu }=\mu_{\max}\times \frac{1}{k_{L}}\times \sum_{i=1}^{k_{L}}\frac{G_{i}(t)}{K_{S}+G_{i}(t)+\frac{G_{i}{\left(t\right)}^{2}}{k_{I}}} \end{array}$$

Equations (8) replace the continuous Equations (1)–(3). The constant *C* = $3600\times 10^{-6}$ is present in Equation (7) because the light intensity (µmol/m^2^/s) will be finally expressed in mol photons/m^2^/h.

We recall that *q*(*t*) is the control input for this process. With a consecrated notation, it holds

*u*(*t*) = *q*(*t*)

The state variables have the following significance: $x_{1}\left(t\right)$ = the biomass concentration; $x_{2}\left(t\right)$ = the amount of light consumed up to moment *t*. The second state variable $x_{2}\left(t\right)$ will be considered within the process model only when the PBR productivity must be evaluated.

In our work, we are interested in two PBR parameters that can be considered as output variables: the specific growth rate and the biomass existing in the PBR. These parameters are calculated as follows:

(9) $$SGR(t)=\bar{\mu }-\mu_{d}$$

(10) $$m(t)=V\times x_{1}(t)$$

*SGR* indirectly depends on *t* through the intermediary of $x_{1}(t)$ and q(t).

(2) Constraints

For the PBROC, the following constraints have to be met:

control horizon: *t*_0_
*≤ t ≤ t_f_*; (11)

initial conditions: *x*(*t*_0_) *= x*_0_ (12)

bound constraints: *q_m_ ≤ q*(*t*) *≤ q_M_*, with *t*_0_
*≤ t ≤ t_f_*. (13)

The constants *t*_0_ and *t_f_* are the initial and final times of the control horizon; *q_m_* and *q_M_* are, respectively, the minimum and maximum values of the admissible light intensity.

Generally speaking, a dynamic system’s evolution might be subjected to other types of constraints: path constraints, terminal equality constraints, algebraic equality, etc.

(3) Optimum criterion

In our context, the optimal control seeks the control input u(·) minimizing an objective function *I*. We recall hereafter its continuous general form:

$$\;I(u,t_{0},t_{f})=\int_{t_{0}}^{t_{f}}L\left(x\left(t\right),u\left(t\right)\right)dt+M\left(t_{f},x_{f}\right)$$

The first part is a Lagrange-type term that measures the quality along the trajectory of the dynamic system; the second part is a Mayer-type term that measures the quality of the trajectory in its final state.

The proposed OCP involves finding out the control variable *u*(·) that meets all constraints and minimizes the objective function *I*. In this paper, a value $I(u,t_{0},t_{f})$ is associated with each evolution of the system (Equations (6) and (7)):

(14) $$I=\alpha \times x_{2}(t_{f})+\beta {\left(m(t_{f})-m_{0}\right)}^{2}$$

The constant *m*_0_ is an imposed value for the biomass *m*(*t*) at the final moment *t_f_*. The value $x_{2}(t_{f})$ is the total amount of light that has radiated the microalgae culture. Our OCP has the optimum criterion *J* defined below:

(15) $$J=\underset{u}{\min}\;I(u,t_{0},t_{f})$$

Equations (14) and (15) correspond to a *bilocal optimization problem* with a fixed final time. The minimization of the first term corresponds to a small amount of light, while a small value for the terminal penalty means achieving the goal *m*_0_. Taking into account the second state equation from Equations (7) and (15), we can render the optimum criterion in the form

(16) $$J=\underset{q(t),\;t_{0}\leq t\leq t_{f}}{\min}\left[w_{1}\times \int_{t_{0}}^{t_{f}}q(t)\times dt+w_{2}{\left(m(t_{f})-m_{0}\right)}^{2}\right].$$

The constants *w*_1_ and *w*_2_ are the new scale factors, whose setting is an important issue. These constants must sufficiently penalize the non-fulfilment of the bilocal constraint but avoid the minimization of the light term falling into eclipse.

For PBR users, it is important to have minimal final biomass, that is

(17) $$m(t_{f})\geq m_{0}.$$

This constraint regards the state variable $x_{1}$ because it can be written as follows:

(18) $$x_{1}(t_{f})\geq \frac{m_{0}}{V}.$$

**Remark** **1.** *The control algorithm (containing the Predictor module) will generate a set of system trajectories. The constraint in Equation (17) can be seen as a path constraint defining the admissible trajectories.*

Adding the inequality in Equation (17) to the set of constraints in Equations (11)–(13), we render the optimum criterion in its final form:

(19) $$J=\underset{q(t),\;t_{0}\leq t\leq t_{f}}{\min}\left[w_{1}\times \int_{t_{0}}^{t_{f}}q(t)\times dt+w_{2}\left(m(t_{f})-m_{0}\right)\right].$$

Therefore, we have a weighted combination of the two criteria that transforms our OCP into a standard optimization problem with a single optimal criterion. To summarize, the PBROC can be seen as a procedure with:

- Input data: Equations (5)–(13), (17) and (19), all the PBR constructive parameters and constants;
- Output data: the optimal control variable $q(t),\;t_{0}\leq t\leq t_{f}$.

**Remark** **2.** *In the following, PM (from process model) denotes Equations (5)–(13). The PBROC problem is described through the PM plus Equations (17) and (19).*

NB: This work aims to give a closed-loop solution to the PBROC problem, which is more complex than finding the optimal control input sequence applied from the known initial state.

### 3.2. Receding Horizon Control Structure

For the PBROC problem stated before, we need a closed-loop solution, i.e., an optimal controller that sends a value *q*(*t*), *t*_0_ ≤ *t* ≤ *t_f_* toward the BPR in each sampling period. The current value *q*(*t*) will be computed based on the current state of the BPR such that the process will meet all the constraints (Equations (5)–(13) and (17)) and the optimum criterion (Equation (19)).

**Figure 2:**
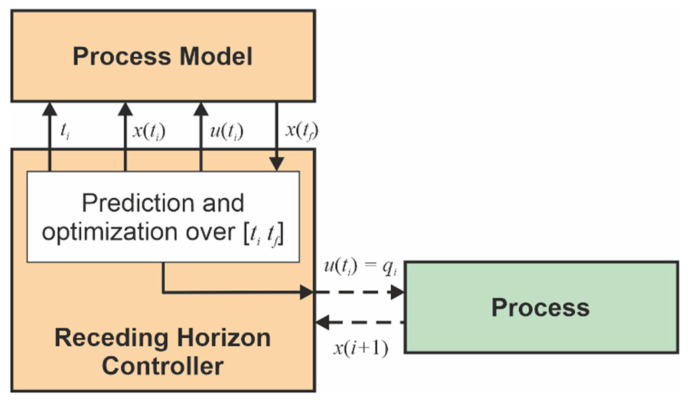
Closed loop with Receding Horizon Control.

The control structure adopted in this work to achieve the closed-loop solution is RHC (see [16,17]) as presented in Figure 2. RHC involves the calculation of the optimum criterion over the interval $\left[t_{k}\;t_{f}\right]$, where $\left[t_{k}\;t_{k+1}\right]$ is the current sampling period. Therefore, the controller has to predict the future evolution of the process (see [16]). That is why the RHC structure includes a process model, which allows predictions to be made using the current real state x(k) acquired from the process. The controller selects its control output u(k) (for the process, it is the control input) as the prediction’s first element and sends it toward the process. The process feedback arrives after a sampling period when the new state x(k+1) is achieved.

### 3.3. Controller’s Predictions

In the following, we use the discrete form of the PBROC problem (Equations (5)–(13), (17) and (19)) in a way that is adapted to the presentation of the next sections. The control horizon is finite [0, H×T], where *H* is a positive integer and *T* is the sampling period. The discrete moments $t_{k}=k\times T$ will be specified simply by k=0,1,…,H.

In this work, we consider a realistic case of the control input q(t), which is constant within a sampling period *T*:

(20) $$q(t)=q_{i},\;t\in [i\times T,\;(i+1)\times T);\;\mathrm{where}\;i=0,\ldots ,H-1;\;t_{f}=H\times T.;\;q_{m}\leq q_{i}\leq q_{M}.$$

Hence, the control input is a step function. The continuous process model (Equations (5)–(9)) can always be converted into a discrete one as follows:

(21) x(k+1)=f(k,x(k),u(k)); k=0, 1,…, H−1,

where u(k)=q(t), $x(k)={\left[x_{1}(k)\;x_{2}(k)\right]}^{T}$, and *f* is a two-dimensional vector function.

Any overall control sequence $Q_{0}$ meets all constraints and determines the system evolution over the control horizon. It is defined as follows:

(22) $$Q_{0}\overset{D}{=}<q(0),\;q(1),\ldots ,\;q(H-1)>.$$

The optimum criterion (Equation (19)) has its discrete form presented hereafter.

(23) $$J=\underset{Q_{0}|{}_{m(H)\geq m_{0}}}{\min}\left\{w_{1}\times A\times C\sum_{i=0}^{H-1}q(i)+w_{2}\times \left[V\times x_{1}(H)-m_{0}\right]\right\}.$$

The min operator is applied for the set of sequences that leads to final biomass greater or equal to $m_{0}$.

At each moment *k*, the controller makes predictions over the predicted horizon [k, H]. A predicted control sequence has the following form:

(24) $$pcs(k)\overset{D}{=}<q(k|k),\;q(k+1|k),\ldots ,\;q(H-1|k)>,\;k=0,\;1,\ldots ,\;H-1,$$

where q(k+i|k), i=0,…,H−k−1 is the predicted value for the control input q(k+i) based on our knowledge up to moment k.

The controller uses an algorithm to generate such control sequences and to determine the optimal one. In this work, the optimal predictions will be made by the APSO algorithm.

**Remark** **3.** *Note that the state x(k) is not estimated because it is acquired from the process at every sampling moment. This state, the process model (Equation (21)), and a generated pcs(k) allow estimation of all the intermediary states and computation of the objective function.*

The control input q(k+i|k) is kept constant within the sampling period [*k* + *i*, *k* + *i* + 1], such that a *pcs* represents a step function.

**Remark** **4.** *It should also be noted that q(k+i|k)≠q(k+i); the value q(k+i|k) is a future control input predicted at the present moment, whereas the future real control input q(k+i) is unknown at the prediction moment. We can assert the same thing for the state variables.*

Using the current state x(k)=x(k|k), the generated *pcs*(*k*) and the process model (Equation (21)), the controller can calculate the corresponding predicted state sequence, *pss*(*k*), as defined below:

(25) $$pss(k)\overset{D}{=}<x\left(k|k\right),\ldots ,x\left(H\;|k\right)>,\;k=0,\;1,\ldots ,\;H-1$$

Equation (25) refers to the prediction horizon [k,H] and the state trajectory that starts with x(k). The sequence *pss*(*k*) has a length greater by one unit than *pcs*(*k*).

Within the RHC loop, the controller has to make an optimal prediction, that is, to calculate the optimal control sequence (*ocs*)

(26) $$ocs(k)\overset{D}{=}<q^{*}\left(k|k\right),\ldots \;,\;q^{*}\left(H-1|k\right)>,\;k=0,\;1,\ldots ,\;H-1$$

that minimizes the objective function, as below:

(27) $$J(k,x(k))=\underset{pcs(k)|{}_{m(H)\geq m_{0}}}{\min}\left\{w_{1}\times A\times C\sum_{i=k}^{H-1}q(i)+w_{2}\times \left[V\times x_{1}(H)-m_{0}\right]\right\}$$

(28) ocs(k)=arg J(k,x(k))

The optimal state sequence *oss*(*k*) is the *pss*(*k*) corresponding to the optimal control sequence (*ocs*). The controller can now establish the current control output using ocs(k). This is the first element of the sequence ocs(k):

(29) $$u(k)=q^{*}(k|k)\overset{D}{=}q^{*}(k).$$

Table 1 presents an outline of the controller’s actions for every sampling period. Its structure is a general one that uses a generic function (“*Predictor*”).

To determine the optimal prediction in line #2 is not a simple job because of the computational complexity the *Predictor* faces. Except for the first value of the sequence ocs(k), the remaining part is not used. This fact has a simple explanation: *ocs* also proves that an admissible final state is accessible from the current state x(k). The same thing must be proved for the next moment (*k* + 1), starting from a new state variable acquired from the process (Remark 3).

### 3.4. A Soft Sensor to Determine the Range of Control Output Values

#### 3.4.1. Necessity

Generally speaking, the control output value is subjected to the constraint in Equation (13), where the bounds *q_m_* and *q_M_* are mainly technological limits. The light intensity *q*(*k*) can be supplied between these bounds. For example, in this work, *q_m_* = 50 and *q_M_* = 2000 µmol·m^−2^·s^−1^, which is a very large interval. Therefore the computational complexity of the algorithm making the predictions is very important.

We define the admissibility domain ($D_{k}$) of the control input as the interval containing all the values that lead to a favorable process evolution (e.g., meeting certain dynamic constraints). For example, we can consider only the input values that entail positive values for the growth rate of microalgae. We emphasize that the admissibility domain depends on the current state of the process and the “favorable” dynamic constraint at hand.

If the current state of the process entails, for certain physical reasons, a narrower interval $D_{k}\subset \left[q_{m},\;q_{M}\right]$ such that $q(k)\in D_{k}$, this situation could be used to diminish the computational complexity. The process control input will be looked for in a smaller interval. Hence, the constraint in Equation (13) could be replaced by

(30) $$q(k)\in D_{k},\;D_{k}\subset \left[q_{m},\;q_{M}\right].$$

NB: The process control input may be considered, at the same time, to be the control output q(k) issued out from the controller because it is the connection between the controller and the process.

Using narrower intervals, we expect to improve the Predictor’s computational complexity, which in this simulation study is based on the APSOA. This improvement will be proved in the next sections.

This section proposes a soft sensor to reduce the admissibility domain of the process’s control input according to its current state. The sensor returns information about the domain $D_{k}$ meeting Equation (30). This is based on:

- measurement of the biomass concentration ($x_{1}(k)$) and
- the numerical integration of the PM over the next sampling period ([k,k+1]) for a certain number of light intensity values. These values are considered successively as the control output for the current sampling period:

(31) $$q_{0}(l),\;l=1,\ldots ,nl;\;q_{0}(l)\in [q_{m},\;q_{M}];$$

For example, in our simulations, we have adopted *nl =* 40.

The sensor’s estimations are based on a real measure $x_{1}(k)$ and refer only to the current sampling period to be realistic. In the next sampling period, the sensor will be based on the new real measure $x_{1}(k+1)$.

The sensor makes the integrations and generates its output OUT(*k*), which the *Predictor* can use in two different ways to define a smaller interval $D_{k}$ (as described later in this section).

#### 3.4.2. Controller Structure with Soft Sensor

**Figure 3:**
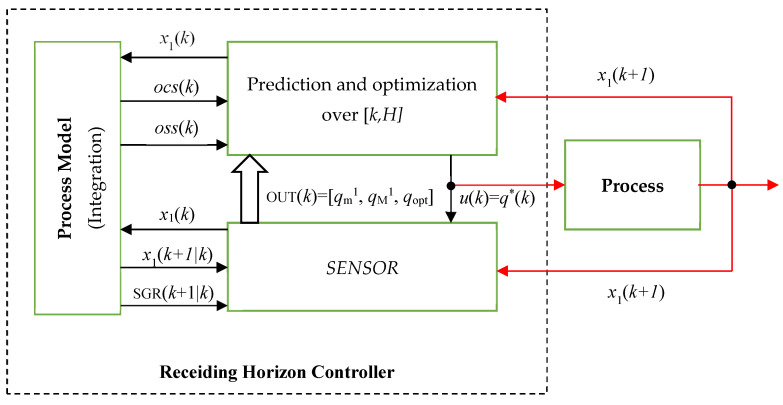
Receding Horizon Controller with sensor.

Figure 3 shows how the *SENSOR* is included in the controller. It also shows how data circulate among modules and between the controller and the process in sampling period *k*. To understand this figure, let us suppose that the biomass concentration $x_{1}(k)$ is acquired from the process at the beginning of the current sampling period. All the modules, including the *SENSOR*, have the data necessary for their calculations and estimations. Finally, the controller computes the output *u*(*k*) = q*(*k*), which is sent toward the process (red lines are used for the information exchanged between the controller and the process).

The latter evolves due to the control output *u*(*k*) and will produce a new biomass concentration returned to the controller after a sampling period ($x_{1}(k+1)$).

#### 3.4.3. Output Parameters of the Soft Sensor

To understand what the soft sensor evaluates and what information is returned, we shall analyze the PBR evolution over a period of time, for example, [0, 50] hours.

**Figure 4:**
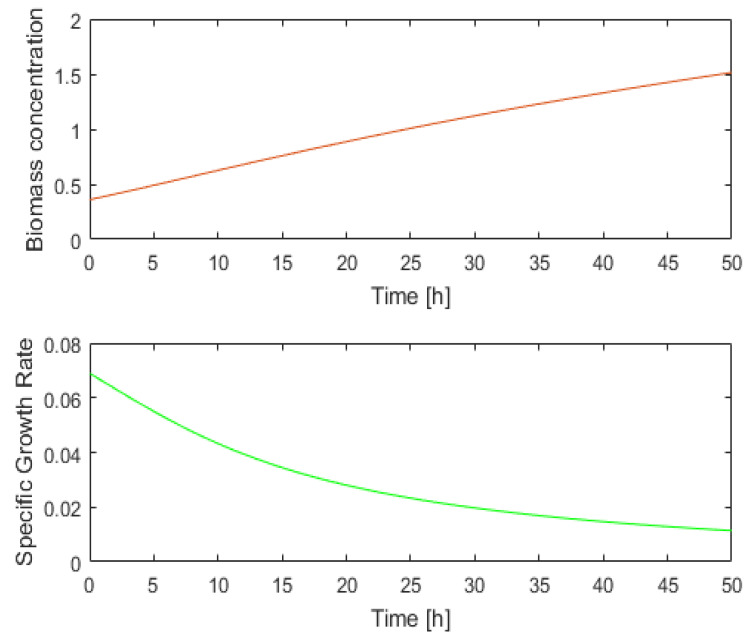
Evolution of *x*_1_(*t*) and SGR(*t*) over 50 h.

Figure 4 depicts the evolution of the state variables during the interval [0, 50] hours. The initial values of the state variables and all the system parameters are presented in Appendix A. At the moment *k* = 50, the following values are obtained through numerical integration of the PM:

(32) $$x_{1}(50)=\;1.5137\;g/L\;SGR(50)=0.0114{\;h}^{-1}.$$

**Figure 5:**
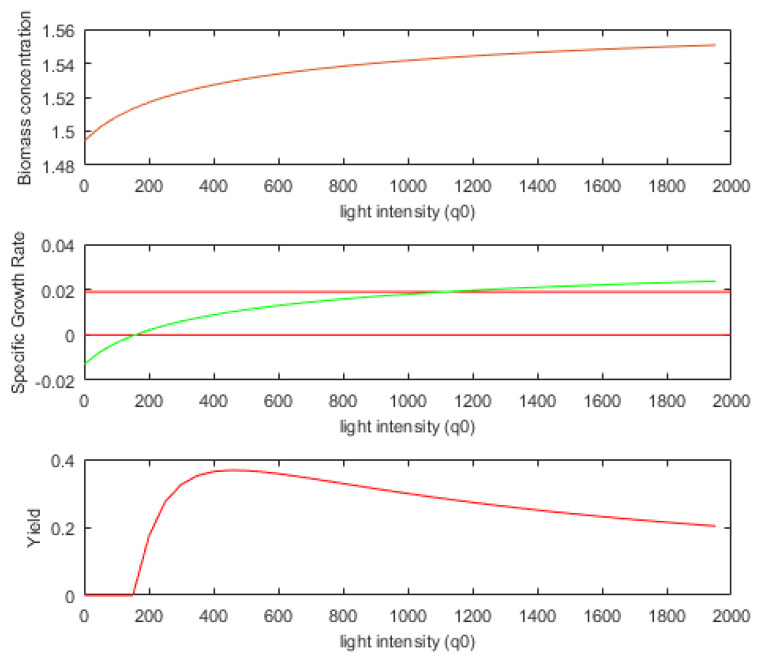
Dependence of biomass concentration, SGR, and biomass yield on light energy as a function of light intensity.

One may wonder if there is a narrower interval, *D*(50), within which the controller would search for the next control output *u*(50). To respond to this question, a certain number of values for light intensity *u*(50) = *q*(50) = $q_{0}$ can be tested. Considering the initial state (Equation (32)), we carried out the numeric integration of the PM over the next sampling period (one hour) for all these values (see Equation (31), with *nl* = 40). We noted the final values for the biomass concentration, *SGR*, and biomass yield on light energy. Figure 5 presents these final values as a function of light intensity.

The soft sensor returns information that can define narrower intervals $D_{k}\subset \left[q_{m},\;q_{M}\right]$ for the current sampling period. We can call this interval the admissibility domain for the control output at moment *k*. There are two modes of using this information.

In the first mode (*mode* = 1), the light intensity, *q*(*k*), avoids the values placed at the extremities of the interval $\left[q_{m},\;q_{M}\right]$. On the left, the values for which the specific growth is negative are avoided because the biomass will decrease. On the other side, very large light intensity values lead to growth saturation inside the PBR and a certain inefficiency when considering biomass yield on light energy. In this mode, the light intensity belongs to the following interval:

(33) $$\begin{array}{l} D(k)=[q_{m}^{1},\;q_{M}^{1}];\\ q_{m}^{1}=\min\{q_{0}|SGR(q_{0})\geq 0\};\\ q_{M}^{1}=p\times \arg\underset{q_{0}\in [q_{m},\;q_{M}]}{\max}SGR(q_{0});\;0<p<1 \end{array}.$$

The value $q_{M}^{1}$ is a fraction of the light intensity that produces the maximum *SGR*. In this work, we have considered *p =* 0.8. Therefore, in our example, a reasonable choice is to consider D(50)=[150, 1101] µmol·m^−2^·s^−1^, like in Figure 5 where the red lines define the two values ($q_{m}^{1}$ and $q_{M}^{1}$). Aiming to use this mode, the first two output parameters of the soft sensor are $q_{m}^{1}$ and $q_{M}^{1}$.

The second mode of using the sensor (*mode* = 2) is related to the biomass yield on light energy (Equation (4)). Figure 5 shows *Y* as a function of light intensity. In our case, the value $q_{opt}(k)=451$ µmol·m^−2^·s^−1^ determines a maximum value for *Y*. This information can be useful in control applications that aim to maximize *Y* over larger intervals. In the PBROC problem, apparently, this information would not be useful because we have to minimize the amount of light while a specified quantity of biomass is produced. Nevertheless, the strategy to control the PBR using light intensity values around the optimal value ($q_{opt}$) would have a beneficial influence. In this sensor mode, the light intensity belongs to the following interval:

(34) $$D(k)=[(1-r)\times q_{opt}(k),\;(1+r)\times q_{opt}(k)];\;0<r<1.$$

In this work, after a few tests, we chose the value r=0.2, which offers a sufficient range for the control output and keeps a certain optimal behavior. The simulations proved that this mode produces very good results. Let us remark that if the value of *Y* is near optimal in the current sampling period, the amount of light is near minimal for the newly produced biomass. This is why the global optimum criterion can be determined using the interval in Equation (34).

The soft sensor’s third parameter is the value $q_{opt}(k)$. It belongs to the calling application to compute the extremities of the interval in Equation (34) using an appropriate value *r*. Finally, the list of parameters returned by the soft sensor is

(35) $$OUT\left(k\right)=\left[q_{m}^{1}\left(k\right),q_{M}^{1}\left(k\right),q_{opt}\left(k\right)\right].$$

According to one of the two modes described above, it is up to the calling application to use this information.

#### 3.4.4. Implementation of the Soft Sensor

The *SENSOR* function is described by the pseudocode presented in Table 2. To estimate its output parameters, the *SENSOR* has the biomass concentration as the input variable. Other important information is available through global variables: the current moment, *k*, sampling period, *T*, and the PBR’s dynamic model SPM (simplified to PM). The latter includes only Equations (6) and (9) concerning the biomass concentration and *SGR*.

**Remark** **5.** *The biomass concentration can be determined online due to a linear correlation between the dry matter and the turbidity of the culture. The dry matter can be determined daily, and the turbidity measured online (see [10]).*

After the numerical integration of the SPM, to estimate its output parameters, the *SENSOR* uses very simple and fast methods whose precision is satisfactory. The greater the value of *nl*, the more precisely the three output parameters are determined. A very high precision for these parameters is not mandatory because the two intervals $D_{k}$ will be used in a stochastic environment.

Line #1 can be implemented, for example, by a loop that generates the following vector:

(36) $$q_{0}=[q_{m},\;q_{m}+\Delta ,\;q_{m}+2\Delta ,\ldots ,q_{M}];\;\Delta =\frac{q_{M}-q_{m}}{nl-1}.$$

The numerical integration for the *nl* light intensities is carried out within lines #3–#6. The function *EvalState* is called at line #4 and achieves the SPM numerical integration over a sampling period ([0 *T*]), starting from the initial state $X_{0}$ and applying the light intensity $q_{0}(j)$. The integration results are the biomass concentration (*Bc*(*j*)), *SGR*(*j*), and *Y*(*j*) at the final moment, *T*.

Lines #7–#13 calculate $q_{m}^{1}(k)$ as being the first light intensity that involves a positive value of *SGR.* The value $q_{M}^{1}(k)$ is determined in lines #14–#22 as being $q_{0}(j_{2})$, to which the *SGR* that is the closest to *Sg*80 (defined in line #14) corresponds. The third output parameter is calculated in lines #23–#24. The value $q_{opt}(k)$ ensures the maximum value of *Y*.

A real implementation of the *SENSOR*—using the MATLAB language and system—is presented within the files “SENSOR.m” and “XXEvalState.m” inside the folder “PSO_pred_senz”.

## 4. Prediction Based on Adaptive PSO Algorithm

### 4.1. Brief Description of PSO

PSO is a well-known metaheuristic used in many applications [24,25,26]. To keep the presentation self-contained, we present hereafter a few elements defining this metaheuristic for the readers that are newcomers in the field of PSO. Its detailed structure is given in Appendix B.

A particle swarm system aims to optimize an objective function with *n* decision variables through a simulated movement of the particles within the search space where the objective function is defined. The latter models the “environment” shape, i.e., the altitude of “valleys” and “hills”, which characterizes the optimization problem (OP). Each particle “flies” over new regions and updates its information. In the initial version of PSO, the particles communicate among themselves through the intermediary of a global variable, memorizing the swarm’s best position “encountered”.

As the search process proceeds, the exploration decreases, and the exploitation is intensified. Finally, the particles converge to the global best solution of the OP. The main elements concerning the analysis of PSO algorithms are presented in [24]. The convergence of PSO algorithms is addressed in [23,24]. Results guide the choice of algorithm parameters.

The swarm is composed of *N* particles, coded through a three-component vector, usually denoted by $\left(X_{i},V_{i},P_{best_{i}}\right)$. Each component is an *n*-dimensional vector representing the position, speed, and best personal position reached in the search process (see [20]). It holds as:

$$X_{i}=\left(x_{i}^{1},x_{i}^{2},\ldots ,x_{i}^{d},\ldots ,x_{i}^{n}\right),\;V_{i}=\left(v_{i}^{1},\;v_{i}^{2},\ldots ,\;v_{i}^{d},\ldots ,v_{i}^{n}\right)$$

$$P_{best_{i}}=\left(p_{best_{i}}^{1},\ldots ,p_{best_{i}}^{d}\ldots ,p_{best_{i}}^{n}\right),\;i=1,\;2,\;\ldots ,\;N$$

An iterative process simulates the evolution of the particle swarm. $P_{gbest}$ denotes the particles’ best position up to the current step, called the “global best” position. When the algorithm ends, that is when convergence is achieved; $P_{gbest}$ is the OP solution.

The PSO algorithm’s main action is updating the speed and position of particles at each iteration of the search process. This update is carried out using the following equations:

(37) $$\{\begin{array}{l} V_{i}\left(t+1\right)=w\times V_{i}\left(t\right)+C_{1}\times rand_{1}\times \left(P_{best}(t)-X_{i}(t)\right)+C_{2}\times rand_{2}\times \left(P_{gbest}(t)-X_{i}(t)\right)\\ X_{i}(t+1)=X_{i}(t)+V_{i}(t+1) \end{array}$$

*w* is inertia weight, *C*_1_ and *C*_2_ are acceleration coefficients, *rand*_1_ and *rand*_2_ are random numbers in the interval [0, 1], and *t* is the step number.

### 4.2. Adaptive PSO Algorithm

The PSO algorithm used in this paper uses two advances: hybrid topology and adaptation of particle speed. Hybrid topology particle swarm optimization (HTPSO) [19,24] improves the PSO metaheuristic with better communication abilities among particles. It is also enhanced with adaptive updating of the particle speed by a continuous change of some algorithm parameters.

The proposed algorithm will be referred to as APSOA (Adaptive PSO Algorithm), implicitly including hybrid topology.

APSOA also uses a swarm’s local topology, regarded as a communication network. The local topology means the existence, for any particle #*i*, of a “social neighborhood”, i.e., a set of 3–5 particles that inform particle #*i* about their best personal experience. These neighborhoods are settled deterministically or randomly (see [19,24]). Each particle will decide the local best position $P_{lbest_{i}}$, which is the best experience of the particles belonging to the “social neighborhood” (including the particle itself). It holds that:

(38) $$P_{lbest_{i}}=\left(p_{lbest_{i}}^{1},\ldots ,p_{lbest_{i}}^{d}\ldots ,p_{lbest_{i}}^{n}\right);\;d=1,\ldots ,\;n;$$

A new term, containing *C*_3_ and *rand*_3_, appears in the speed equation (see [24]). The updating of the speed and position is performed using the following equations:

(39) $$\{\begin{array}{l} \begin{array}{ll} v_{i}^{d}\left(t+1\right) & =w\times v_{i}^{d}\left(t\right)+C_{1}\times rand_{1}\times \left(p_{best_{i}}^{d}(t)-x_{i}^{d}(t)\right)+\\ & +C_{2}rand_{2}\left(p_{lbest_{i}}^{d}(t)-x_{i}^{d}(t)\right)+C_{3}rand_{3}\left(p_{gbest}^{d}(t)-x_{i}^{d}(t)\right) \end{array}\\ x_{i}^{d}(t+1)=x_{i}^{d}(t)+v_{i}^{d}(t+1) \end{array};\;d=1,\ldots ,n$$

An efficient technique can enhance the APSOA: adaptation of particle speed. This technique modifies the coefficients *C*_1_, *C*_2_, *C*_3_, and *w* during the iterative process (see [19]). These coefficients are adapted to the phase of the search process and prepared to help the algorithm’s convergence. A linear increase for the coefficients *C*_1_, *C*_2_, and *C*_3_ between their minimum and maximum values will adapt the particles’ speed. At the same time, the parameter *w* decreases as follows:

(40) $$\begin{array}{l} C_{1}\left(t\right)=C1min+\left(C1max-C1min\right)t/T;\;C_{2}\left(t\right)=C2min+\left(C2max-C2min\right)t/T;\\ C_{3}\left(t\right)=C3min+\left(C3max-C3min\right)t/T;\;w\left(t\right)=wmax-\left(wmax-wmin\right)\times t/T \end{array}$$

*T* is the estimated or maximum number of steps until convergence.

Because the structure of APSOA is well known and has already been presented in many papers, including [19,24], this structure is described in Table A1 in Appendix B. However, some specific characteristics are mentioned hereafter:

- APSOA is organized like a function of five input parameters;
- The predicted sequence length (*h*) is an input parameter because RHC involves predicted sequences with different lengths;
- *k* and $x_{0}$ define the moment and initial state of the process, for which the optimal sequence is determined.
- $x_{m}$ and $x_{M}$ are vectors with *h* elements. They are composed of the minimum and maximum values for each component of the predicted sequence. The values $x_{m}(1)$ and $x_{M}(1)$ are determined by the *SENSOR*. The other values are set to $q_{m}$ and $q_{M}$ respectively.
- The function *RHC_EvalFitnessJ* evaluates the objective function resulting from Equation (27).

### 4.3. Predictor Structure

The structure of the *Predictor* is presented in Table 3 as the pseudocode of the function *Predictor_SZ*, which has the following input parameters:

- k is the current moment when the controller calls the Predictor to make an optimal prediction;
- $x_{0}(k)$ is the initial state (biomass concentration) acquired from the process;
- $x_{m}^{1}$ and $x_{M}^{1}$ are the two extremities of the interval $D_{k}$ determined by the *SENSOR* (whatever the utilization mode).

The predicted sequence will have a variable dimension, *h*, according to line #2, where *n* corresponds to the total control horizon.

The Predictor has two main tasks:

- To set the two vectors’ values, the control output limits for the predicted sequences (lines #3–#6). The two vectors ($x_{m}$ and $x_{M}$) have the same length as the predicted sequences. The values from the *SENSOR* are assigned to the first element of these vectors, corresponding to the first control output $u^{*}(k)$. The other limits are $q_{m}$ and $q_{M}$.
- To call the function APSOA with the appropriate arguments. The best prediction (*ocs*) is returned to the controller.

To describe the *Predictor*’s structure simply, APSOA was presented before as a function (a separate program unit). In our real implementation, the APSOA code is included in the *Predictor* code. Details are given in Appendix B.

## 5. Simulation Study

The organization of similar simulations is presented in [27,28,29]. All of them concern applications of computational intelligence [30].

### 5.1. Study Objectives and Preliminaries

All the control tools developed in previous sections are available now to tackle the PBROC problem stated in Section 3.1. This simulation study has a few objectives, as listed below:

1. To implement and simulate a closed loop based on Receding Horizon Control devoted to solving the PBROC problem.
2. To study the feasibility of the prediction technique using a metaheuristic algorithm. In our case, we have chosen the Adaptive PSO Algorithm. When metaheuristic algorithms are involved in optimal control, the main impediment often encountered is the computational complexity, which affects the controller’s computing time for the control output.
3. To validate the hypothesis that the computational complexity of the prediction will diminish by introducing the admissibility domain $D_{k}$ for the control output.
4. To validate the hypothesis that the proposed soft sensor can realistically provide the admissibility domains using the biomass concentration measured from the process.

Objectives 1 and 2 will be analyzed together as well as objectives 3 and 4 because they are intrinsically connected.

The simulation study will be performed with an application that emulates the closed-loop functioning. Some modules will be implemented realistically, like in a real-time application, with only a few adjustments devoted to the simulation.

- The Controller, which by definition includes the PM, is also connected to the real process (PBR). The soft sensor is connected to the process as well. Both modules are realistically implemented but should have connections with the process (PBR). Only these connections are simulated.
- In our application, the real process is also simulated using the PM (see Remark 2). The red lines in Figure 3 also show the simulated connections that create the closed loop.
- The sequence of sampling moments is simulated.

### 5.2. Closed-Loop Implementation

The algorithm *RHC_Closed_Loop*, which simulates the closed-loop solution of the PBROC problem and will allow us to analyze the proposed tools, is described in Table 4. 

After the appropriate initializations for the PM, APSOA, and simulation, the algorithm is mainly structured through a “while-loop” that follows the sampling moments $k=0,\ldots ,t_{f}-1$.

Basically, this loop uses the *SENSOR* (lines #10–#17), calls the *Predictor* (line #18), sets the control output (line #19), obtains the new state of the process (line #20), and moves on to the next sampling moment (lines #21–#23).

The variable *mode,* set in line #2, specifies how the *SENSOR* is used. If *mode* = 0, *the SENSOR* is not called, and the admissibility domain $D_{k}$ is the widest possible (#11). The *SENSOR* is called within line #12, as though *mode* = 1. If it is actually *mode* = 2, lines #13–#15 make the corrections.

The function “$\mathrm{RealProcessStep}(u(k),x_{0},k)$” simulates the state evolution of the real process. It integrates the PM over the next sampling period, starting from the initial state $x_{0}$ and having the step function u(k) as a control input.

Lines #19 and #20 contain comments corresponding to the red lines from Figure 3. The vector “*state*” has (*n* + 1) elements and stores the process states for k=0,1,…,n. Finally, it represents the global *oss* (see Section 3.3) obtained with RHC.

Details concerning the implementation of the *RHC_Closed_Loop* algorithm are given in Appendix C.

## 6. Results

### 6.1. Simulation of the Tandem SENSOR–Predictor

To better understand how the *SENSOR* and *Predictor* modules work together, we present in this section a simulation of their standalone execution (which is not included in the closed-loop control). We calculate the optimal control sequence at the moment *k* = 0, that is, an *ocs* with n = 120 control output, considering different values for $m_{0}$. The *SENSOR* (*mode* = 2) is called before the *Predictor* call as below:

$$\left[x_{m}^{1},x_{M}^{1},u_{opt}\right]\leftarrow SENSOR(x_{0},\Delta t)$$

$$x_{m}^{1}\leftarrow (1-p)*u_{opt};\;x_{M}^{1}\leftarrow (1+p)*u_{opt}$$

$$\mathrm{ocs}\leftarrow \mathrm{Predictor}\_\mathrm{SZ}(k,\;x_{0},\;x_{m}^{1},\;x_{M}^{1}).$$

This relates to lines #12–#18 of the *RHC_Closed_Loop* algorithm (Table 4). Considering the initial biomass concentration $x_{1}(0)=0.36$ g/L, the PM is integrated using *ocs*. Table 5 presents the results.

For each execution, five values are displayed: the target $m_{0}$ (g), newly produced biomass (g), value of the optimum criterion (*J*), amount of Light $x_{2}(H)$ (mol photons/m^2^/h), and the number of calls (Ncalls) of the objective function. We can conclude that the target $m_{0}$ is precisely achieved in a small number of objective function calls.

### 6.2. Closed-Loop Simulation without SENSOR

In this section, the simulation results of the RHC structure for solving the PBROC problem are presented. We give hereafter the input data for the problem we have solved.

The PM presented in Section 3.1 is characterized by the physical and constructive parameters given in Appendix A. In addition, we have the following initial data:

- Control horizon: 120 h; $t_{f}=120\;h$; n=H=120;
- Sampling period: 1 h; *T* = 1;
- Light intensity bounds: $q_{m}=50;\;q_{M}=2000$ (µmol·m^−2^·s^−1^);
- Initial biomass concentration: $x_{1}(0)=0.36$ g/L.

Desiderata:

- To fulfil the optimum criterion in Equation (27), where $w_{1}=1;\;w_{2}=10$;
- To ensure the newly produced biomass:
  m_0_ ≥ 3 g (41)

The APSOA has the parameters presented in Appendix B and tuned for this application. Their values result clearly from the first lines of the script RHC_PSO_PBRJ13_SZ.m. Very important for the algorithm’s computational complexity is the number of adopted particles in the swarm:

*N* = 15 (42)

Because the prediction is based on a stochastic algorithm, to analyze the simulation results, the *RHC_Closed_Loop* program was ran 30 times for each instance of the PBROC problem. Practical details about this operation are given in Appendix C.1.

A realistic measure of this application’s computational complexity is the number of calls of the objective function during the control horizon. This fact is true, especially when the objective function involves numerical integrations, which have significant complexity and are time-consuming. This measure is also adequate for comparison between different versions of applications. That is why the *RHC_Closed_Loop* totalizes the number of calls for each sampling period.

The results of this simulation series without *SENSOR* (controller in *mode* = 0) are given in Table 6.

For each execution, four values are displayed: the value of the optimum criterion (*J*), the amount of light (Light) over the entire control horizon (mol photons/m^2^/h), newly produced biomass (g), and the average number of calls (Ncalls). The latter equals the total number of calls divided by the number of sampling periods (*n* = 120). The sampling periods have very different numbers of calls: the greater the value of *k*, the smaller the number of calls. However, it is easier to perceive and compare the total number of calls divided by 120 (Ncalls).

**Remark** **6.** *It can be seen that newly produced biomass equals almost exactly 3 g for all executions. This fact is the consequence of how the constraint in Equation (41) is checked. Equation (27) defining the optimum criterion over the prediction horizon [k,H] specifies that only the predicted control sequences that lead to $m(H)\geq m_{0}$ are considered. In our implementation of the objective function (like a programming unit), the constraint in Equation (41) is treated like a trajectory constraint (Remark 1). If the constraint in Equation (41) is not met, the value returned by the objective function is infinity, and the trajectory is not admissible. Otherwise, it equals the sum of the two terms (see file RHC_EvalFitnessJ13.m, which implements the objective function). On the other side, the optimum criterion in Equation (27) looks for a minimum for both terms and their sum. The smaller the amount of light, the smaller the value $m(H)-m_{0}$ (for physical reasons). Therefore, naturally, APSOA will find a minimum for which we have $m(H)-m_{0}\approx 0$.*

Because the constraint in Equation (41) is met for all the executions, the produced biomass cannot discriminate among the quality of simulations. Therefore, we shall determine the typical one among the 30 simulations using, of course, the value of the optimum criterion. We shall consider the simulation that produces the closest value to the average optimum criterion as the typical execution.

The minimum, average, and maximum values and standard deviation for the optimum criterion (*J*) are given in Table 7. 

In our simulation series, there is an execution that yields *J*_typical_ = 9.226. The other simulation results (line #29 in Table 6) are presented hereafter:

$$J_{\mathrm{typical}}=9.226;\;\mathrm{Light}=9.226;\;\Delta m=3.0000;\;\mathrm{NCalls}=798.25$$

**Figure 6:**
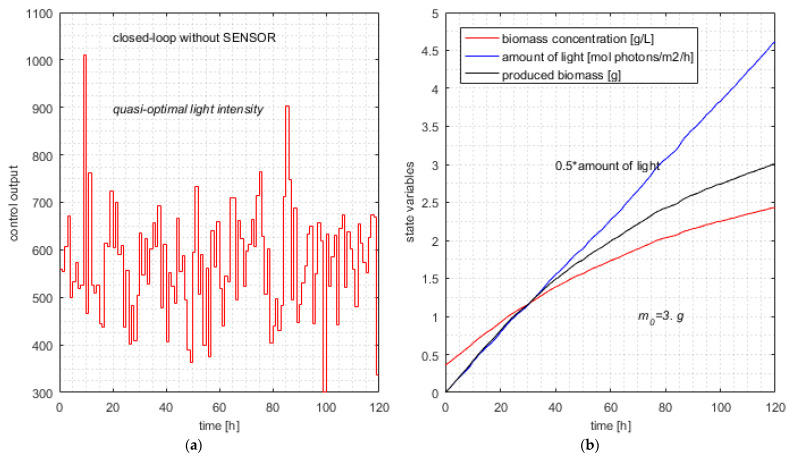
Typical simulation of the closed loop without soft sensor. (**a**) Typical control output without SENSOR; (**b**) quasi-optimal evolution of state variables and produced biomass in the typical execution.

For each execution, the 120 values of the quasi-optimal control output are recorded for the final simulation of the closed loop. The light intensity for the typical execution is depicted in Figure 6a.

This control action involves the typical evolution of the state variables in Equations (6) and (7) and the newly produced biomass depicted in Figure 6b.

The simulations have proved that the APSOA converges for all executions. This fact is a characteristic of this metaheuristic with the considered advances. Despite the computational complexity of the first sampling period, when the *pcs*’s length is maximal, the controller calculates the control variable in a few decades of seconds (depending on processor speed). This amount of time is very satisfactory for a sampling period of 1 h. Hence, the control structure could be a solution even for real-time control.

### 6.3. Closed-Loop Simulation Using the Soft Sensor (mode = 1)

The *RHC_Closed_Loop* program with SENSOR (*mode* = 1) was ran 30 times for the same instance of the PBROC problem as in the previous section. Practical details about this simulation are given in Appendix C.2. The results of this simulation series are given in Table 8.

Table 8 has two particularities. The first one concerns column Δ*m*, which always equals exactly 3 g. The second one is the consequence of the first one; columns *J* and Light are identical because the second term of *J* is practically null. The constant $w_{2}$ determines these particularities because it privileges the minimization of *J*’s second term.

**Remark** **7.** *A smaller value of $w_{2}$ entails a relaxation for the minimization of the second term; finally, the value of J will be greater, and the values of J and Light will be different. This relaxation will lead to a relaxation of the searching process and, consequently, a smaller number of objective function calls. Hence, we have a spare strategy to diminish the number of calls, choosing a smaller value of $w_{2}$. The price to pay is greater light and a greater newly produced biomass (to a small extent).*

Table 9 presents the new statistics for the optimum criterion (*J*). The average value is practically the same as in Table 6, but what is noteworthy is the standard deviation, which is smaller by 43%. The new values of *J* are positioned in a smaller interval. The difference between *J*_max_ and *J*_min_ equals 0.292, smaller than 0.484 as in Table 6. This is the consequence of the controller’s commands, which belong to smaller intervals $D_{k}$.

The typical simulation produces the following results (line #23 in Table 8):

$$J_{\mathrm{typical}}=9.271;\;\mathrm{Light}=9.271;\;\Delta m=3.0000;\;\mathrm{NCalls}=661.375.$$

The average value of Ncalls from Table 8 is 734.

**Figure 7:**
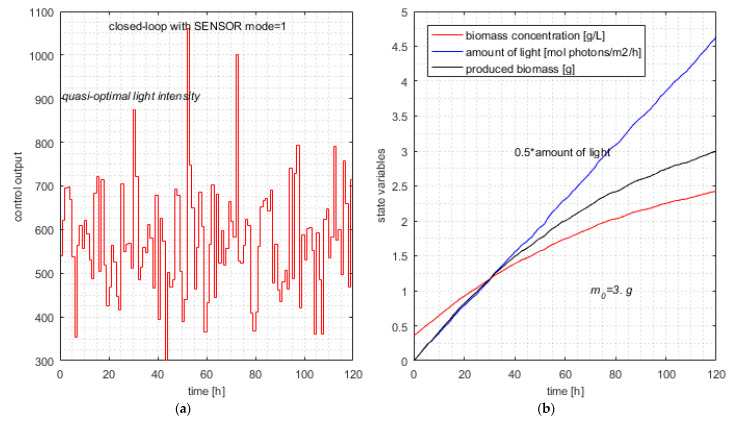
Typical simulation of the closed loop with *SENSOR mode* = 1. (**a**) Typical control output—controller with metaheuristic-based predictions; (**b**) quasi-optimal evolution of state variables and produced biomass.

The typical simulation of the closed loop with *SENSOR* (controller in *mode* = 1) is illustrated in Figure 7.

**Remark** **8.** *The average number of calls of the objective function equals 734, which is smaller than 791 by 7.2%. This decrease is expected, and although not spectacular, it proves the improvement in computational complexity due to the soft sensor. The efficiency of the soft sensor is confined to a certain extent because it has a limited action. At moment k, the pcs covers the interval [k, H] and has the form given by Equation (24). The soft sensor improves the bounds only for the first component pcs(k); the other H-k-1 elements will have the initial bounds given by Equation (13). The SENSOR’s estimations are based on a real measure $x_{1}(k)$ and could not be extended to the other pcs components because the next real states are unknown. The estimations refer to the current sampling period to keep them realistic.*

**Remark** **9.** *The increase in computation complexity is negligible when using the SENSOR because the PM’s integration covers only the current sampling period. It should be noted that the controller already acquires the biomass concentration to implement the closed loop.*

### 6.4. Closed-Loop Simulation Using the Soft Sensor (mode = 2)

This time, the *SENSOR* calculates the best biomass yield on light energy for the current sampling period. The RHC_Closed_Loop program with SENSOR (*mode* = 2) was ran 30 times for the same instance of the PBROC problem as in the previous sections. Practical details are given in Appendix C.3 about how the simulation results were generated. The results are presented in Table 10.

Table 10 has only two columns because the light values equal the *J* values and the newly produced biomass is always 3 g, as in Table 7. The minimum, average, maximum and typical values and standard deviation for the optimum criterion (*J*) are given in Table 11.

The typical simulation produces the following results (line #6 in Table 10):

$$J_{\mathrm{typical}}=8.429;\;\mathrm{Light}=8.429;\;\Delta m=3.0;\;\mathrm{NCalls}=660.$$

The average value of Ncalls is 686.8, smaller than 791 from Table 6 (without SENSOR) by 13.1%. This decrease in computational complexity is even more important when we consider the argumentation of Remark 8.

**Figure 8:**
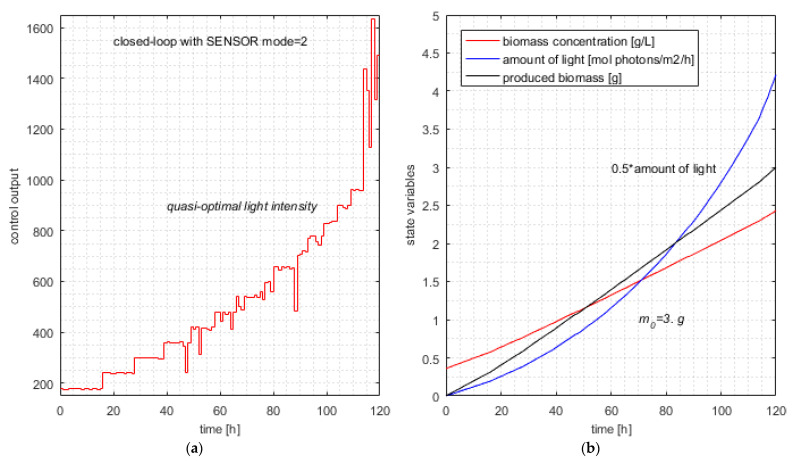
Typical simulation of the closed loop with SENSOR mode = 2. (**a**) Typical control output—controller with metaheuristic-based predictions; (**b**) quasi-optimal evolution of state variables and produced biomass.

The typical simulation of the closed loop with SENSOR (controller in *mode* = 2) is illustrated in Figure 8.

To explain the SENSOR’s good result, we recall that we want to minimize the amount of light on the entire control horizon while the constraint in Equation (41) is fulfilled. This desideratum is equivalent to maximizing *Y* over the entire control horizon while meeting the constraint in Equation (41).

**Remark** **10.** *Maximizing Y in each sampling period does not imply the maximum value of Y over the interval [0, H]. It is a “greedy” strategy that, in general, does not ensure the global optimum. Nevertheless, if the value of Y is near optimal in the current sampling period, the amount of light is near minimal in relation to the newly produced biomass. At the same time, the predicted sequence guarantees that the constraint in Equation (41) is met because it generates an admissible trajectory.*

After a few exploratory tests of Equation (34), we chose the value r=0.2, which offers a sufficient range for the control output to face the minimization of *J*.

**Remark** **11.** *The appearance of Figure 8, which entails a certain monotony of the control output, is due to the monotony of the value $q_{opt}$ as a function of initial biomass concentration, which is considered by the sensor within its estimations.*

## 7. Conclusions

For the PBR and its PM, which is well known and has been validated in previous papers, we have stated the PBROC problem. The implementation of its solution requires metaheuristic-based predictions. The Adaptive PSO Algorithm was chosen for its convergence speed. Despite the ability of APSOA to efficiently generate optimal predictions (in a reasonable time), we have proposed a soft sensor to cope with a very large prediction horizon (e.g., 120). 

The main contribution of our work is the soft sensor, which was conceived to diminish the Predictor’s computational complexity. The *SENSOR* is based on measurement of the biomass concentration and numerical integration of the process model over the current sampling period. The returned information concerns the specific growth rate of microalgae and the biomass yield on light energy. Our proposal generated two modes of using the soft sensor to reduce the admissibility intervals (*D*(*k*)) for the process control input (*u*(*k*)).

The simulation study proved that our main contribution, the *SENSOR,* reduced the computational complexity significantly, expressed by the number of calls of the objective function.

To conduct the simulation study presented in this paper, we implemented the proposed algorithms using the MATLAB language and system. The Supplementary Materials (attached to this article) include scripts and files that show how the closed loop, Controller, *SENSOR,* and *Predictor* (including the APSOA) can be implemented with good results. Many algorithms and their implementation could be used in a future real-time application, at least as a starting point.

For future work, we could investigate the conception of sensors for other processes as a general idea, aiming for the same objective of reducing the admissibility intervals. The difficulty would be in disclosing the biophysical aspects that justify the limitation of the admissibility domains according to the current state of the process.

## Figures and Tables

**Table 1:** Outline of Receding Horizon Controller.

1	Obtain the current value of the state vector, *x*(*k*).
2	ocs(k)←arg J(k,x(k))/*call *Predictor* function*/
3	$u(k)\leftarrow q^{*}(k)$
4	Send u(k) towards the dynamic system
5	Shift the prediction horizon and wait for the next sampling period

**Table 2:** Algorithm of the soft sensor.

	**function**$\left[q_{m}^{1}(k),\;q_{M}^{1}(k),\;q_{opt}(k)\right]$ ← $\mathrm{SENSOR}(x_{1}(k))$
1	Initialize the light intensity vector $\left(q_{0}(l),\;l=1,\ldots ,nl\right)$ according to Equation (31).
2	Initializations: $p=0.8;\;X_{0}=[x_{1}(k)\;0].$
3	**for***j* = 1,…,*nl*
4	[Bc(j),SGR(j),Y(j)]← *EvalState*(SPM, $X_{0},\;[0,T],\;q_{0}(j)$)
5	**if** Y(j)<0 then Y(j)=0
6	**end**
7	$found\leftarrow 0;\;j_{1}\leftarrow 1$ /*start computing $q_{m}^{1}(k)$*/
8	**while** $(j_{1}<nl)\;\&\;(\mathrm{found}=0)$
9	**if** $(SGR(j_{1})>0)\;found\leftarrow 1$
10	**else** $j_{1}\leftarrow j_{1}+1$
11	**end**
12	**end**
13	$q_{m}^{1}(k)\leftarrow q_{0}(j_{1})$
14	Sg80←p×max{SGR(j), j=1,…,nl} /* start computing $q_{M}^{1}(k)$*/
15	$j_{2}\leftarrow 1;\;dif\leftarrow \left|SGR(j_{2})-Sg80\right|$;
16	**for** m=2,…,nl
17	dx=|SGR(m)−Sg80|
18	**if** (dx<dif)
19	$dif\leftarrow dx;\;j_{2}\leftarrow m$;
20	**end**
21	**end**
22	$q_{M}^{1}\leftarrow q_{0}(j_{2})$
23	$j_{3}\leftarrow \arg\underset{j}{\max}\{Y(j),\;j=1,\ldots ,nl\}$ /* start computing $q_{opt}(k)$*/
24	$q_{opt}(k)\leftarrow q_{0}(j_{3})$
25	**end** *SENSOR*

**Table 3:** Algorithm of the *Predictor* function.

	**function***Predictor_SZ*(*k*, $x_{0},\;x_{m}^{1},\;x_{M}^{1}$)
1	Initialization; /*space reservation for each particle*/
2	h←n−k
3	$x_{m}(1)=x_{m}^{1};\;x_{M}(1)=x_{M}^{1};$ /*From SENSOR*/
4	**for**i=2,…,h /* if h≥2*/
5	$x_{m}(i)=q_{m};\;x_{M}(i)=q_{M};$ /*Technological limits*/
6	**end**
7	$Pgbest\leftarrow \mathrm{APSOA}(k,\;h,\;x_{0},\;x_{m},\;x_{M}$) /*call the metaheuristic */
8	ocs←Pgbest
9	**return** ocs

**Table 4:** Closed-loop simulation—description of *RHC_Closed_Loop* algorithm.

1	Initializations: PM parameters and constants, see Appendix A Some parameters and constants of the APSOA
2	*mode* = 0; *p* = 0.2; /*or *mode* = 1, or *mode* = 2*/
3	Compute $k_{i},\;i=1,\ldots ,k_{L}$ /*see (5)*/
4	$x_{0}\leftarrow 0.36$; /*Initial biomass concentration*/
5	*state*(1) ← $x_{0}$;
6	n←120; /*Final moment $t_{f}=120$ h*/
7	Δt←1; /*SENSOR’s Horizon */
8	k←0; /*Sampling moment counter*/
9	**while***k* <= *n* − 1
10	**if** (*mode* = 0)
11	$x_{m}^{1}\leftarrow q_{m};\;x_{M}^{1}\leftarrow q_{M}$
12	**else** $\left[x_{m}^{1},x_{M}^{1},u_{opt}\right]\leftarrow SENSOR(x_{0},\Delta t)$ /*mode = 1 or *mode* = 2*/
13	**if** (*mode* = 2)
14	$x_{m}^{1}\leftarrow (1-p)*u_{opt};\;x_{M}^{1}\leftarrow (1+p)*u_{opt}$
15	**end**
16	**end** /*else*/
17	**end** /*if*/
18	*ocs* ← *Predictor_SZ*(*k*, $x_{0},\;x_{m}^{1},\;x_{M}^{1}$)
19	u(k)←*ocs*(1); /* optimal control output $q^{*}(k)$*/ /*Send u(k) towards the process */
20	$x_{next}\leftarrow \mathrm{RealProcessStep}(u(k),x_{0},k)$; /*Wait for the next sampling moment and obtain the next state of the process*/
21	$x_{0}\leftarrow x_{next}$ /* the new initial state */
22	k←k+1 /*next sampling period */
23	*state*(*k* + 1) ← $x_{0}$
24	**end** /*while*/
25	/*Generate and display simulation results*/

**Table 5:** Results produced by the standalone couple *SENSOR*–*Predictor*.

$m_{0}$	Δm	*J*	$x_{2}(H)$	NCalls
1.5	1.5012	3.3389	3.3273	210
1.8	1.8013	3.8872	3.8747	240
2.0	2.0005	4.5031	4.4984	195
2.2	2.2018	5.6522	5.6344	300
2.4	2.4035	6.2092	6.1744	180
2.6	2.6166	7.6403	7.4739	180
2.8	2.8926	9.8765	8.9504	330
3.0	3.0019	8.9459	8.9267	240

**Table 6:** Simulation series for *RHC_Closed_Loop* without SENSOR.

Run #	*J*	Light $\left[\frac{mol\;photons}{m^{2}\cdot h}\right]$	Δm (g)	NCalls	Run #	*J*	Light $\left[\frac{mol\;photons}{m^{2}\cdot h}\right]$	Δm (g)	NCalls
1	9.0136	9.0077	3.0006	960.00	16	9.2717	9.2717	3.0000	672.625
2	9.0423	9.0347	3.0008	665.000	17	9.3212	9.3212	3.0000	727.250
3	9.0523	9.0523	3.0000	951.000	18	9.2314	9.2314	3.0000	689.000
4	9.0448	9.0447	3.0000	845.000	19	9.2276	9.2276	3.0000	758.125
5	9.1961	9.1838	3.0012	940.000	20	9.2726	9.2726	3.0000	731.125
6	8.9792	8.9764	3.0003	962.000	21	9.2753	9.2753	3.0000	848.500
7	9.1024	9.0582	3.0044	663.000	22	9.3992	9.3992	3.0000	717.375
8	9.0728	9.0691	3.0004	959.000	23	9.2007	9.2007	3.0000	738.750
9	9.0605	9.0419	3.0019	841.000	24	9.2302	9.2302	3.0000	839.875
10	9.0059	8.9988	3.0007	941.000	25	9.4145	9.4145	3.0000	654.000
11	9.3527	9.3527	3.0000	773.625	26	9.4045	9.4045	3.0000	745.500
12	9.4327	9.4327	3.0000	662.875	27	9.2029	9.2029	3.0000	759.125
13	9.2569	9.2569	3.0000	747.750	28	9.4126	9.4126	3.0000	753.625
14	9.2506	9.2506	3.0000	785.125	29	9.2264	9.2264	3.0000	798.250
15	9.4629	9.4629	3.0000	812.375	30	9.2924	9.2924	3.0000	790.500

**Table 7:** Statistics regarding the optimum criterion.

*J*_min_	*J*_avg_	*J*_max_	S_dev_	*J*_typical_
8.979	9.224	9.463	0.142	9.226

**Table 8:** Simulation series for *RHC_Closed_Loop* with SENSOR *mode* = 1.

Run #	*J*	Light $\left[\frac{mol\;photons}{m^{2}\cdot h}\right]$	Δm (g)	NCalls	Run #	*J*	Light $\left[\frac{mol\;photons}{m^{2}\cdot h}\right]$	Δm (g)	Ncalls
1	9.2035	9.2035	3.0	773.00	16	9.1755	9.1755	3.0	740.875
2	9.1904	9.1904	3.0	717.00	17	9.3622	9.3622	3.0	735.625
3	9.2315	9.2315	3.0	788.00	18	9.1986	9.1986	3.0	678.500
4	9.4021	9.4021	3.0	739.00	19	9.3125	9.3125	3.0	739.625
5	9.1366	9.1366	3.0	728.00	20	9.3089	9.3089	3.0	728.750
6	9.3324	9.3324	3.0	715.00	21	9.2234	9.2234	3.0	639.125
7	9.2815	9.2815	3.0	784.00	22	9.3419	9.3419	3.0	791.875
8	9.2265	9.2265	3.0	779.00	23	9.2711	9.2711	3.0	661.375
9	9.1967	9.1967	3.0	791.00	24	9.2027	9.2027	3.0	818.875
10	9.1718	9.1718	3.0	763.00	25	9.2818	9.2818	3.0	744.875
11	9.3391	9.3391	3.0	854.00	26	9.2994	9.2994	3.0	639.500
12	9.2078	9.2078	3.0	686.375	27	9.1633	9.1633	3.0	764.875
13	9.1327	9.1327	3.0	690.625	28	9.4251	9.4251	3.0	690.125
14	9.2975	9.2975	3.0	765.375	29	9.2925	9.2925	3.0	660.00
15	9.3036	9.3036	3.0	742.125	30	9.3970	9.3970	3.0	676.00

**Table 9:** Statistics regarding the optimum criterion (*SENSOR mode* = 1).

*J*_min_	*J*_avg_	*J*_max_	S_dev_	*J*_typical_
9.133	9.264	9.425	0.080	9.271

**Table 10:** Simulation series for *RHC_Closed_Loop* with SENSOR *mode* = 2.

Run #	*J*	NCalls	Run #	*J*	Ncalls
1	8.4403	704.250	16	8.4083	677.500
2	8.4640	638.000	17	8.3669	688.625
3	8.4104	612.000	18	8.4294	660.000
4	8.5084	648.875	19	8.4403	704.250
5	8.3898	715.000	20	8.4640	638.000
6	8.4294	660.000	21	8.4104	612.000
7	8.4403	704.250	22	8.5084	648.875
8	8.4560	768.750	23	8.3898	715.000
9	8.4576	810.125	24	8.3952	730.500
10	8.4159	740.625	25	8.3973	630.625
11	8.4429	728.375	26	8.3669	672.125
12	8.4224	724.125	27	8.4253	777.000
13	8.4655	702.375	28	8.4120	570.000
14	8.3984	714.750	29	8.4475	717.000
15	8.4156	648.000	30	8.4803	645.625

**Table 11:** Statistics regarding the optimum criterion (*SENSOR* mode = 2).

*J*_min_	*J*_avg_	*J*_max_	S_dev_	*J*_typical_
8.367	8.430	8.508	0.036	8.429

## Supplementary Materials

The following are available online at https://www.mdpi.com/article/10.3390/s21238065/s1, The archive file “PSO_pred_senz.zip” contains all files described in Appendix B and Appendix C.

## Appendix A

### Details Concerning the Photobioreactor

- The radiative model: the absorption coefficient, $E_{a}$ = 172 m^2^·kg^−1^; the scattering coefficient, $E_{s}$ = 870 m^2^·kg^−1^; the backward scattering fraction, b = 0.0008 (-).
- The kinetic model: the specific growth rate, $\mu_{max}$ = 0.16 h^−1^; the saturation constant, $K_{S}$ = 120 µmol·m^−2^·s^−1^; the inhibition constant, $K_{I}$ = 2500 µmol·m^−2^·s^−1^; the specific decay rate, $\mu_{d}$ = 0.013 h^−1^.
- Volume of the reactor, V = 1.45 × 10^−3^ m^3^; the depth, L = 0.04 m; the lighted surface, A = 3.75 × 10^−2^ m^2^.
- The initial biomass concentration $x_{1}(0)=0.36$ g/L.

The simulation in Section 3.4.3 was carried out using $q_{0}=502.3$ μmol/m^2^/s (the light intensity that radiates the PBR).

The mathematical model was validated (see [10]) on microalgae strains such as *Chlamydomonas reinhardtii* and *Scenedesmus quadricauda* (see [9]) cultivated in laboratory flat-plate photobioreactors in similar conditions. The biomass concentration, measured offline as dry mass, can be correlated with turbidity, measured online by a sensor. The PBRs are lighted on one side by dimmable LED panels, which the process computer can control. The calibration of the LED panel can be done with a portable light meter; the incident light intensity is measured inside the reactor at z = 0.

## Appendix B

### Description of APSOA and Implementation of Predictor

**Table A1:** Description of adaptive particle swarm optimization algorithm.

	**function**$Pgbest\leftarrow \mathrm{APSOA}(k,\;h,\;x_{0},\;x_{m},\;x_{M}$)
1	*Input parameters*: *k*—the current discrete moment; $x_{0}$—the current state (biomass concentration); *h*—predicted sequence length ($X_{i}$ has *h* elements) $x_{m}$: vector with *h* elements—minimum value for each component $x_{M}$: vector with *h* elements—maximum value for each component
2	Initialization: *N*, *h*, v_max_,* x*_min_,* x*_max_,* C1*_min_,* C1*_max_,* C2*_min_,* C2*_max_,* C3*_min_,* C3*_max_,* w*_min_,* w*_max_,* T*, *NF*, *MINNF*
3	Generate the particles’ initial velocities as uniformly distributed values in the interval [−v_max_, v_max_]*^h^*.
4	Generate the particles’ initial positions $X_{i},\;i=1,\ldots ,N$;
5	**for** i=1,…,N
6	$P_{best_{i}}\leftarrow X_{i}$
7	BEST(*i*), *fitness(i)* ← *RHC_EvalFitnessJ*$(X_{i},\;x_{0}$, *k*)
8	**end**
9	*i*_0_← arg max {BEST(*i*), *i* = 1,…,*N*};
10	$P_{gbest}\leftarrow X_{i_{0}}$; GBEST ← BEST(*i*_0_); /*Determine $P_{gbest}$ and GBEST */
11	*found* ← 0
12	*t* ← 1;
13	**while** (*t* <= *T*) & (*found* = 0)
14	Coefficients tuning: *w,* *C*_1_, *C*_2_, *C*_3_
15	**for** i=1,…,N /* move the particles swarm*/
16	$P_{lbest}{}_{{}_{i}}$ ← *Generate_Plbest*(*i*); /* generate “local best”*/
17	**for** d=1,…,n
18	Update the particles’ speed using Equation (39)
19	*Speed_limitation*$(v_{i}^{d}\left(t+1\right)$)
20	$x_{i}^{d}\left(t+1\right)=x_{i}^{d}\left(t\right)+v_{i}^{d}\left(t+1\right)$
21	*Position_ limitation* $\left(x_{i}^{d}\left(t+1\right)\right)$ /*using $x_{m},\;x_{M}$*/
22	**end**
23	*fitness(i)* ← *RHC_EvalFitnessJ*$(X_{i},\;x_{0}$, *k*)
24	**if** *fitness*(*i*) < BEST(*i*)
25	$P_{best}{}_{{}_{i}}\leftarrow X_{i}$; BEST(*i*) ← *fitness*(*i*); *NF*(*i*) = 0;
26	**else** *NF*(*i*) = *NF*(*i*) + 1;
27	**end**
28	**if** *fitness*(*i*) < GBEST(*i*)
29	$P_{gbest}\leftarrow X_{i}$; GBEST ← *fitness*(*i*)
30	**end**
31	**if** *min* {*NF*(i), *i* = 1,…,*N*} = *MINNF* **then** *found* ← 1;
32	*t* ← *t* + 1
33	**end** /*while */
34	**return** *Pgbest*

The APSOA code is included in the Predictor code in our implementation, which is the script “RHC_Predictor_SZ.m”. The objective function is implemented within the file “RHC_EvalFitnessJ13.m”. Both files are inside the folder “PSO_pred_senz”.

## Appendix C

The general algorithm *RHC_Closed_Loop* was implemented according to the three employment modes and generated three applications (three scripts). Each of them calls the function “RHC_RealProcessStep.m”.

All the scripts are included in the folder “PSO_pred_senz”, attached to this work.

### Appendix C.1. Simulation Details for RHC_Closed_Loop without SENSOR (mode = 0)

The algorithm *RHC_Closed_Loop* was implemented by the script “RHC_PSO_PBRJ13withoutSZ.m”. This program was ran 30 times using the script “Loop30_8_50_withoutSZ.m”. The computation of the statistical parameters, which are presented in Table 5 and Table 6, was conducted using the script “MEDIERE30loop_withoutSZ.m”. Figure 6 was generated using the script “DRAWfigWithoutSZ.m”.

### Appendix C.2. Simulation Details for RHC_Closed_Loop with SENSOR (mode = 1)

For this mode, the algorithm RHC_Closed_Loop was implemented by the script “RHC_PSO_PBRJ13_SZ.m”. This program was ran 30 times using the script “Loop30_8_50_SZ.m”. The computation of the statistical parameters, which are presented in Table 7 and Table 8, was conducted using the script “MEDIERE30loop_cuSZ.m”. Figure 7 was generated using the script “DRAWfigCuSZ.m”.

### Appendix C.3. Simulation Details for RHC_Closed_Loop with SENSOR (mode = 2)

For this mode, the algorithm RHC_Closed_Loop was implemented by the script “RHC_PSO_PBRJ13_SZv2.m”. This program was ran 30 times using the script “Loop30_8_50_SZv2.m”. The computation of the statistical parameters, which are presented in Table 9 and Table 10, was conducted using the script “MEDIERE30loop_cuSZv2.m”. Figure 8 was generated using the script “DRAWfigCuSZv2.m”.

## References

[1] Suparmaniam U., Lam M.K., Uemura Y., Lim J.W., Lee K.T., Shuit S.H. (2019). Insights into the microalgae cultivation technology and harvesting process for biofuel production: A review. Renew. Sustain. Energy Rev..

[2] Li X., Liu J., Chen G., Zhang J., Wang C., Liu B. (2019). Extraction and purification of eicosapentaenoic acid and docosahexaenoic acid from microalgae: A critical review. Algal Res..

[3] Acién Fernández F.G., Fernández Sevilla J.M., Molina Grima E. (2013). Photobioreactors for the production of microalgae. Rev. Environ. Sci. Biotechnol..

[4] Ifrim G.A., Titica M., Barbu M., Boillereaux L., Cogne G., Caraman S., Legrand J. (2013). Multivariable feedback linearizing control of Chlamydomonas reinhardtii photoautotrophic growth process in a torus photobioreactor. Chem. Eng. J..

[5] Ifrim G.A., Titica M., Cogne G., Boillereaux L., Legrand J., Caraman S. (2014). Dynamic pH Model for Autotrophic Growth of Microalgae in Photobioreactor: A Tool for Monitoring and Control Purposes. AIChe J..

[6] Jayaraman S.K., Rhinehart R.R. (2015). Modeling and Optimization of Algae Growth. Ind. Eng. Chem. Res..

[7] Cornet J.-F., Dussap C.-G. (2009). A Simple and Reliable Formula for Assessment of Maximum Volumetric Productivities in Photobioreactors. Biotechnol. Prog..

[8] Pottier L., Pruvost J., Deremetz J., Cornet J.F., Legrand J., Dussap C.G. (2005). A fully predictive model for one-dimensional light attenuation by Chlamydomonas reinhardtii in a torus photobioreactor. Biotechnol. Bioeng..

[9] Ifrim G.A., Titica M., Deppe S., Frahm B., Barbu M., Caraman S. Multivariable Control Strategy for the Photosynthetic Cultures of Microalgae. Proceedings of the 23rd International Conference on System Theory, Control and Computing, ICSTCC.

[10] Ifrim G.A., Titica M., Boillereaux L., Caraman S. Feedback Linearizing Control of Light-to-Microalgae Ratio in Artificially Lighted Photobioreactors. Proceedings of the 12th IFAC Symposium on Computer Applications in Biotechnology, CAB.

[11] Tebbani S., Titica M., Ifrim G., Caraman S. Control of the Light-to-Microalgae Ratio in a Photobioreactor. Proceedings of the 18th International Conference on System Theory, Control and Computing, ICSTCC.

[12] Grognarda F., Akhmetzhanov A.R., Bernard O. (2014). Optimal strategies for biomass productivity maximization in a photobioreactor using natural light. Automatica.

[13] Hurst T., Rehbock V. (2008). Optimal control for micro-algae on a raceway model. Biotechnol. Prog..

[14] Park M. (2020). Surface Display Technology for Biosensor Applications: A Review. Sensors.

[15] Andriukonis E., Celiesiute-Germaniene R., Ramanavicius S., Viter R., Ramanavicius A. (2021). From Microorganism-Based Am-perometric Biosensors towards Microbial Fuel Cells. Sensors.

[16] Minzu V., Serbencu A. (2020). Systematic procedure for optimal controller implementation using metaheuristic algorithms. Intell. Autom. And Soft Comput..

[17] Mayne D.Q., Michalska H. (1990). Receding Horizon Control of Nonlinear Systems. IEEE Trans. Autom. Control..

[18] Siarry P. (2016). Metaheuristics.

[19] Talbi E.G. (2009). Metaheuristics—From Design to Implementation.

[20] Kennedy J., Eberhard R. Particle Swarm Optimization. Proceedings of the IEEE International Conference on Neural Networks.

[21] Minzu V., Barbu M., Nichita C. A Binary Hybrid Topology Particle Swarm Optimization Algorithm for Sewer Network Discharge. Proceedings of the 19th International Conference on System Theory, Control and Computing (ICSTCC).

[22] Abraham A., Jain L., Goldberg R. (2005). Evolutionary Multiobjective Optimization—Theoretical Advances and Applications.

[23] Valadi J., Siarry P. (2014). Applications of Metaheuristics in Process Engineering.

[24] Kennedy J., Eberhart R., Shi Y. (2001). Swarm Intelligence.

[25] Maurice C. (2005). L’Optimisation par Essaims Particulaires-Versions Paramétriques et Adaptatives.

[26] Beheshti Z., Shamsuddin S.M., Hasan S. (2015). Memetic binary particle swarm optimization for discrete optimization problems. Inf. Sci..

[27] Minzu V. Quasi-Optimal Character of Metaheuristic-Based Algorithms Used in Closed-Loop—Evaluation Through Simulation Series. Proceedings of the 6th International Symposium on Electrical and Enlectronics Engineering (ISEEE).

[28] Minzu V., Riahi S., Rusu E. (2021). Optimal control of an ultraviolet water disinfection system. Appl. Sci..

[29] Minzu V., Riahi S., Rusu E. (2021). Implementation aspects regarding closed-loop control systems using evolutionary algorithms. Inventions.

[30] Kruse R., Borgelt C., Braune C., Mostaghim S., Steinbrecher M. (2016). Computational Intelligence—A Methodological Introduction.

