# Supplementary figures and images for: Control of Microalgae Growth in Artificially Lighted Photobioreactors Using Metaheuristic-Based Predictions
Source: Sensors (Basel). 2021 Dec 2;21(23):8065. doi: 10.3390/s21238065 (PMC8659673; doi:10.3390/s21238065)

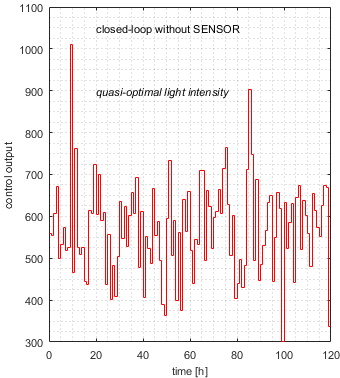

Supplement: Supplementary file 1 [file sensors-21-08065-s001.zip › PSO_pred_senz/FIG 61.png]

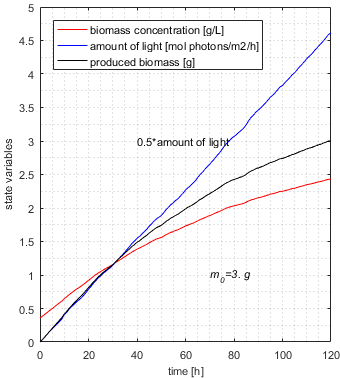

Supplement: Supplementary file 1 [file sensors-21-08065-s001.zip › PSO_pred_senz/FIG 62.png]

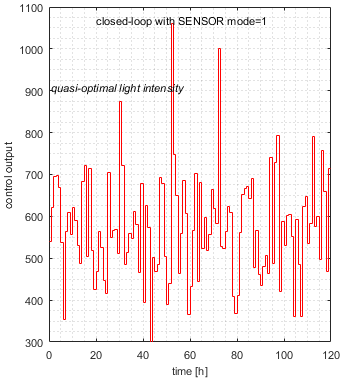

Supplement: Supplementary file 1 [file sensors-21-08065-s001.zip › PSO_pred_senz/FIG 71.png]

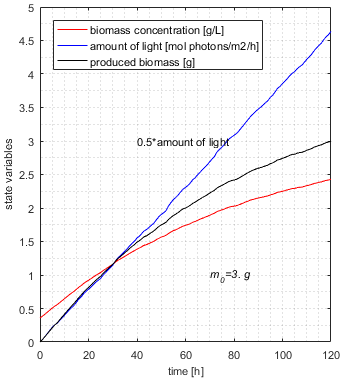

Supplement: Supplementary file 1 [file sensors-21-08065-s001.zip › PSO_pred_senz/FIG 72.png]

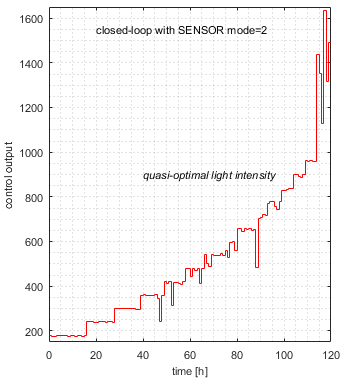

Supplement: Supplementary file 1 [file sensors-21-08065-s001.zip › PSO_pred_senz/FIG 81.png]

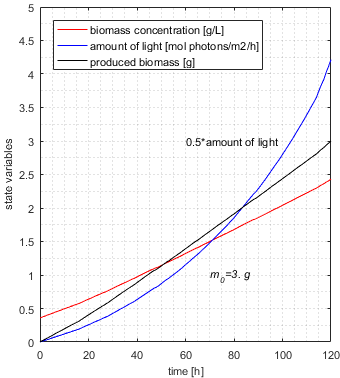

Supplement: Supplementary file 1 [file sensors-21-08065-s001.zip › PSO_pred_senz/FIG 82.png]
